# Supplementary material for: Target cell cortical tension regulates macrophage trogocytosis
Source: Nat Cell Biol. 2025 Dec 12;27(12):2078–88. doi: 10.1038/s41556-025-01807-6 (PMC12716991; doi:10.1038/s41556-025-01807-6)
Supplement: Supplementary file 1 — Supplementary Note. [file 41556_2025_1807_MOESM1_ESM.pdf]

nature cell biology

Supplementary information https://doi.org/10.1038/s41556-025-01807-6

Target cell cortical tension regulates macrophage trogocytosis

In the format provided by the authors and unedited

Supplementary Note

Membrane mechanics scaling for trogocytosis

Here we present details and lay out the assumptions that go into deriving the scaling relationship given in the main text. We assume that deformability is mainly controlled by the target properties, though this assumption can be relaxed to include the composite properties of the macrophage and target membranes.

We start by assuming that there is local binding of the target membrane to the macrophage that is FcR-antibody mediated. For a sufficiently high density of antibody, macrophage binding will trigger downstream signaling machinery that leads to cytoskeleton-mediated active stresses at the interface. These are due, in part, to nucleation of branched actin at the region of binding that drives a height-dependent exclusion of tall bystander proteins and their associated inhibitory phosphatases (*21*). Prior measurements have shown that these active stresses take the form of compressive and extensile normal stresses that deform the interface (*17*). Here we also assume that the magnitude of these stresses is dependent on the antibody density on the target, because more antibody molecules can bind more FcR per unit area and hence drive increased local active stress. Active deformation of the macrophage is resisted by the cortical tension of the target cell. One can therefore derive a scaling relationship by balancing active stresses and cortical tension.

Consider a local deformation of scale R_{min}, due to active stresses (Extended Data Figure 9). Integrating the effects of active stresses and cortical tension gives equation (1):

$$(1) \quad \int_A \sigma_{normal}\, dA \sim \int_S \gamma_t\, ds$$

Where $A \sim R_{min}^2$ is the area over which the normal stresses act, while $S \sim R_{min}$ is the contour length over which tension resists the deformation. A critical scale for these deformations will therefore be equation (2):

$$(2) \quad R_{min} \sim \frac{\gamma_t}{\sigma_{normal}(\rho_{AB})}.$$

This is the minimum length scale of deformation below which tension dominates and will damp out all active fluctuations. Deformations at or above this scale will have the opportunity to grow in amplitude and hence eventually lead to pinch-off of bits of target membrane.

The dependence of σ_{normal} on the local antibody density is unknown, but one can make a reasonable assumption of its form. Below a critical density no engagement must occur between the macrophage and target, and hence no active stresses develop at the interface. Above a critical density we expect the active stresses to be non-zero and also scale with the antibody density and eventually saturate. Based on this, we assume the functional form of equation (3):

$$(3) \quad \sigma_{normal}(\rho_{AB}) = \sigma_{max}\frac{\rho_{AB}}{\rho_{AB} + \rho_{bind}},$$

Where $\sigma_{max} \approx 150$ Pa, is the maximum scale of normal stresses measured in experiments (*17*), and ρ_{bind} is the critical antibody density below which no interaction occurs between the macrophage and target. Based on our experiments we find that this density is ~100 μm^2.
One can similarly derive a second length scale which depends on the target membrane's resistance to local bending deformations due to active stresses, by balancing moments at the interface in equation (4):

$$(4) \quad \sigma_{normal}(\rho_{AB})R_{bend}^2 \sim \frac{\kappa_t}{R_{bend}},$$

where κ_t is the bending rigidity of the target membrane. This leads to the length scale of bend deformations in equation (5):

$$(5) \quad R_{bend} \sim \left[\frac{\kappa_t}{\sigma_{normal}(\rho_{AB})}\right]^{\frac{1}{3}}.$$

This length scale can again be interpreted as a lower threshold below which membrane bending rigidity would preclude deformation. For typical values of bilayer bending stiffness $\kappa_t \approx 400\ k_B T$ and the scale of normal stresses measured at the macrophage-target interface $\sigma_{normal} \approx 100\ Pa$, this equation leads to a predicted deformation scale ~ 0.1 μm. For typical values of tension measured in experiments γ_t ~ 10^{-2} – 10^{-1} mN/m, this means that the tension-mediated minimum length scale is greater and hence controls the scale of the deformations.

We use the above scaling laws to interpret our experimental data as follows. When $R_{min} \gg R$, where R is the total interface size, the macrophage is unable to locally deform the target to pinch-off bites, and we predict that this biases the macrophage towards phagocytic behaviors. On the other hand, if $R_{min} \ll R$, there exists multiple length scales below the interface size that can be deformed and eventually pinched-off by the macrophage. We thus predict that when $R_{min} \ll R$, there is a bias towards trogocytic behaviors. Based on this one can derive a critical tension/antibody density that separates these distinct behaviors by setting $R_{min} \sim R$. Which gives equation (6):

$$(6) \quad \gamma_t|_{crit} \sim \sigma_{normal}(\rho_{AB})R.$$

For a typical macrophage-target interface scale of $\sim 1\ \mu m$ and $\sigma_{normal}(\rho_{AB}) \sim 50 - 150\ Pa$. This gives a critical target cortical tension that separates these behaviors of:
$\gamma_t|_{crit} \sim\ 0.1\ mN/m$.
