# Supplementary material for: Target cell cortical tension regulates macrophage trogocytosis
Source: Nat Cell Biol. 2025 Dec 12;27(12):2078–88. doi: 10.1038/s41556-025-01807-6 (PMC12716991; doi:10.1038/s41556-025-01807-6)
Supplement: Supplementary file 2 — Reporting Summary [file 41556_2025_1807_MOESM2_ESM.pdf]

nature portfolio

Corresponding author(s): Daniel A. Fletcher

Last updated by author(s): Sep 24, 2025

Reporting Summary

Nature Portfolio wishes to improve the reproducibility of the work that we publish. This form provides structure for consistency and transparency in reporting. For further information on Nature Portfolio policies, see our Editorial Policies and the Editorial Policy Checklist.

Statistics

For all statistical analyses, confirm that the following items are present in the figure legend, table legend, main text, or Methods section.

n/a	Confirmed	
☐	☒	The exact sample size (*n*) for each experimental group/condition, given as a discrete number and unit of measurement
☐	☒	A statement on whether measurements were taken from distinct samples or whether the same sample was measured repeatedly
☐	☒	The statistical test(s) used AND whether they are one- or two-sided<br>*Only common tests should be described solely by name; describe more complex techniques in the Methods section.*
☒	☐	A description of all covariates tested
☐	☒	A description of any assumptions or corrections, such as tests of normality and adjustment for multiple comparisons
☐	☒	A full description of the statistical parameters including central tendency (e.g. means) or other basic estimates (e.g. regression coefficient) AND variation (e.g. standard deviation) or associated estimates of uncertainty (e.g. confidence intervals)
☒	☐	For null hypothesis testing, the test statistic (e.g. F, t, r) with confidence intervals, effect sizes, degrees of freedom and P value noted<br>*Give P values as exact values whenever suitable.*
☒	☐	For Bayesian analysis, information on the choice of priors and Markov chain Monte Carlo settings
☒	☐	For hierarchical and complex designs, identification of the appropriate level for tests and full reporting of outcomes
☒	☐	Estimates of effect sizes (e.g. Cohen's *d*, Pearson's *r*), indicating how they were calculated

Our web collection on statistics for biologists contains articles on many of the points above.

Software and code

Policy information about availability of computer code

Data collection	To collect flow cytometry data for this manuscript, Attune Cytometric Software was used . To collect image data for this manuscript, Nikon Elements was used.
Data analysis	All data analysis was done using FlowJo (for flow cytometry data), ImageJ or Nikon Elements (image data), and python (to plot collected data). All code to plot figures in the manuscript is available at Github (https://github.com/fletchlab-git/Trogocytosis) and Code Ocean.

For manuscripts utilizing custom algorithms or software that are central to the research but not yet described in published literature, software must be made available to editors and reviewers. We strongly encourage code deposition in a community repository (e.g. GitHub). See the Nature Portfolio guidelines for submitting code & software for further information.

Data

Policy information about availability of data

All manuscripts must include a data availability statement. This statement should provide the following information, where applicable:

- Accession codes, unique identifiers, or web links for publicly available datasets
- A description of any restrictions on data availability
- For clinical datasets or third party data, please ensure that the statement adheres to our policy

All raw data and processed data needed to plot figures is available in an Open Science Framework repository DOI 10.17605/OSF.IO/6FP4V. All processed data

needed to plot figures from the paper is available in a Code Ocean repository. It is also available as a Statistical Source Data file associated with the manuscript. The data required to plot each subfigure is provided in separate sheets of the .xlsx file.

Research involving human participants, their data, or biological material

Policy information about studies with human participants or human data. See also policy information about sex, gender (identity/presentation), and sexual orientation and race, ethnicity and racism.

Reporting on sex and gender	*Use the terms sex (biological attribute) and gender (shaped by social and cultural circumstances) carefully in order to avoid confusing both terms. Indicate if findings apply to only one sex or gender; describe whether sex and gender were considered in study design; whether sex and/or gender was determined based on self-reporting or assigned and methods used. Provide in the source data disaggregated sex and gender data, where this information has been collected, and if consent has been obtained for sharing of individual-level data; provide overall numbers in this Reporting Summary. Please state if this information has not been collected. Report sex- and gender-based analyses where performed, justify reasons for lack of sex- and gender-based analysis.*
Reporting on race, ethnicity, or other socially relevant groupings	*Please specify the socially constructed or socially relevant categorization variable(s) used in your manuscript and explain why they were used. Please note that such variables should not be used as proxies for other socially constructed/relevant variables (for example, race or ethnicity should not be used as a proxy for socioeconomic status). Provide clear definitions of the relevant terms used, how they were provided (by the participants/respondents, the researchers, or third parties), and the method(s) used to classify people into the different categories (e.g. self-report, census or administrative data, social media data, etc.) Please provide details about how you controlled for confounding variables in your analyses.*
Population characteristics	*Describe the covariate-relevant population characteristics of the human research participants (e.g. age, genotypic information, past and current diagnosis and treatment categories). If you filled out the behavioural & social sciences study design questions and have nothing to add here, write "See above."*
Recruitment	*Describe how participants were recruited. Outline any potential self-selection bias or other biases that may be present and how these are likely to impact results.*
Ethics oversight	*Identify the organization(s) that approved the study protocol.*

Note that full information on the approval of the study protocol must also be provided in the manuscript.

Field-specific reporting

Please select the one below that is the best fit for your research. If you are not sure, read the appropriate sections before making your selection.

☒ Life sciences ☐ Behavioural & social sciences ☐ Ecological, evolutionary & environmental sciences

For a reference copy of the document with all sections, see nature.com/documents/nr-reporting-summary-flat.pdf

Life sciences study design

All studies must disclose on these points even when the disclosure is negative.

Sample size	No sample-size calculation was performed. For measurements of populations of macrophages, approx. 100,000 cells per experimental condition per biological replicate were analyzed. For each experiment, three biological replicates were performed on three different days.
Data exclusions	No data was excluded from the manuscript.
Replication	All experiments were repeated at least three times on three different days to ensure reproducibility. Variation is reflected in the error bars present on figures in the manuscript and described in figure captions. All attempts at replication were successful.
Randomization	This is not relevant to our study as we were investigating cell behavior in cultured immortalized cell lines. Experiments done with bone marrow derived macrophages were performed on cells derived from one mouse.
Blinding	Investigators were not blinded during data acquisition and analysis. As the analytical methods employed require little-to-no subjective input from the investigators, this did not seem necessary.

Reporting for specific materials, systems and methods

We require information from authors about some types of materials, experimental systems and methods used in many studies. Here, indicate whether each material, system or method listed is relevant to your study. If you are not sure if a list item applies to your research, read the appropriate section before selecting a response.

Materials & experimental systems

n/a	Involved in the study
☐	☒ Antibodies
☐	☒ Eukaryotic cell lines
☒	☐ Palaeontology and archaeology
☒	☐ Animals and other organisms
☒	☐ Clinical data
☒	☐ Dual use research of concern
☒	☐ Plants

Methods

n/a	Involved in the study
☒	☐ ChIP-seq
☐	☒ Flow cytometry
☒	☐ MRI-based neuroimaging

Antibodies

Antibodies used: AlexaFluor647-labeled anti-biotin mouse IgG (clone BK-1/39, Santa Cruz Biotechnologies); AlexaFluor647-labeled anti-CD47 mouse IgG (B6H12, Santa Cruz Biotechnologies); anti-CD16/32 mouse IgG (Biolegend)

Validation: Anti-biotin and anti-CD47 were both validated for use in Western Blots or Immunofluorescence. The manufacturer's website provides a list of citations for each antibody as well as details about the validation. Anti-CD16/32 was validated for use as an Fc-receptor blocking antibody through immunofluorescent staining and flow cytometry. A list of citations and information on validation is available on the manufacturer's website.
We used all three antibodies in this study without further purification or labeling.

Eukaryotic cell lines

Policy information about cell lines and Sex and Gender in Research

Cell line source(s): RAW 264.7 cells: Mouse origin, male; Jurkat T cells: Human origin, male; HL60 cells: Human origin, female; Raji B cells: Human origin, male; J774A.1 cells: Mouse origin, female; BMDM cells: Mouse origin, JAX B6 female. M2 cells: Human origin, sex unspecified. All cells except bone marrow derived macrophages were acquired from the Barker Cell Culture Facility at UC Berkeley.

Authentication: All cell lines from the Barker Cell Culture Facility at UC Berkeley are authenticated by the facility.

Mycoplasma contamination: All cell lines tested negative for mycoplasma contamination.

Commonly misidentified lines (See ICLAC register): No cell lines commonly misidentified per ICLAC register were used

Plants

Seed stocks: *Report on the source of all seed stocks or other plant material used. If applicable, state the seed stock centre and catalogue number. If plant specimens were collected from the field, describe the collection location, date and sampling procedures.*

Novel plant genotypes: *Describe the methods by which all novel plant genotypes were produced. This includes those generated by transgenic approaches, gene editing, chemical/radiation-based mutagenesis and hybridization. For transgenic lines, describe the transformation method, the number of independent lines analyzed and the generation upon which experiments were performed. For gene-edited lines, describe the editor used, the endogenous sequence targeted for editing, the targeting guide RNA sequence (if applicable) and how the editor was applied.*

Authentication: *Describe any authentication procedures for each seed stock used or novel genotype generated. Describe any experiments used to assess the effect of a mutation and, where applicable, how potential secondary effects (e.g. second site T-DNA insertions, mosiacism, off-target gene editing) were examined.*

Flow Cytometry

Plots

Confirm that:

☒ The axis labels state the marker and fluorochrome used (e.g. CD4-FITC).

☒ The axis scales are clearly visible. Include numbers along axes only for bottom left plot of group (a 'group' is an analysis of identical markers).

☒ All plots are contour plots with outliers or pseudocolor plots.

☒ A numerical value for number of cells or percentage (with statistics) is provided.

Methodology

Sample preparation	RAW 264.7, J774A.1, or bone marrow-derived macrophages were co-cultured in 96-well TC-treated plates with either HL60s, Jurkat T, Raji B cells, M2 cells, or GUVs. After co-incubation, wells were scraped with miniature cell scrapers, pipette mixed, and immediately analyzed on the flow cytometer.
Instrument	The instrument used was an Attune CytPix Flow Cytometer.
Software	Flow cytometry data was collected using the Attune Cytometric Software. Exported FCS files were then analyzed in FlowJo_v10.8.1.
Cell population abundance	Cells were only analyzed on the flow cytometer, they were not sorted and further experimented upon.
Gating strategy	To determine cells that had trogocytosed or phagocytosed, cells were first gated by FSC and SSC to eliminate small cell debris. Single cells were then selected by drawing a diagonal gate around a SSA vs SSH plot. To isolate macrophages from their target cells, this population was then gated for cells positive for CellTracker Green CMFDA. The populations of cells containing AlexaFluor 647 and/or pHrodo red were then analyzed. Extended Data Figure 8 describes the gating strategy.

☒ Tick this box to confirm that a figure exemplifying the gating strategy is provided in the Supplementary Information.
